# Developing a model for predicting suicide risk among prostate cancer survivors

**DOI:** 10.3389/fmed.2025.1483266

**Published:** 2025-04-10

**Authors:** Jie Yang, Hai-ming Liu, Xiang Qu, Fan Jiang, Jie-wei Hao, Pei-rong Rong, Peng Ning, An-jie Zheng

**Affiliations:** Baoji High-Tech Hospital, Baoji, China

**Keywords:** prostate cancer, suicide risk, nomogram, prevention, SEER

## Abstract

**Objective:**

Given the significantly higher suicide risk among cancer survivors compared to the general population, and considering that prostate cancer survivors make up the largest group of cancer survivors, it is imperative to develop a model for predicting suicide risk among prostate cancer survivors.

**Methods:**

Clinical data of prostate cancer patients were extracted from the surveillance, epidemiology, and end results (SEER) database and randomly divided into a training cohort and a validation cohort in a 7:3 ratio. Initial variable selection was performed using univariate Cox regression, Best Subset Regression (BSR), and Least Absolute Shrinkage and Selection Operator (LASSO). Variables to be included in the final model were selected using backward stepwise Cox regression. Model performance was evaluated using the Concordance Index (C-index), Receiver Operating Characteristic (ROC) curves, and calibration curves.

**Results:**

Data from 238,534 prostate cancer patients were obtained from the SEER database, of which 370 (0.16%) died by suicide. Seven variables including age, race, marital status, household income, PSA levels, M stage, and surgical status were included in the final model. The model demonstrated good discriminative ability in both the training and validation cohorts, with C-indices of 0.702 and 0.688, respectively. ROC values at 3, 5, and 10 years were 0.727/0.644, 0.700/0.698, and 0.735/0.708, respectively. Calibration curves indicated a high degree of consistency between model predictions and actual outcomes. High-risk prostate cancer survivors had a 3.5 times higher risk of suicide than the low-risk group (0.007 vs. 0.002, *P* < 0.001), a finding supported by data from the validation cohort and the entire cohort.

**Conclusion:**

A reliable predictive model for suicide risk among prostate cancer survivors was successfully established based on seven readily obtainable clinical predictors. This model can effectively aid healthcare professionals in quickly identifying high-risk prostate cancer survivors and timely implementation of preventive interventions.

## Introduction

Prostate cancer is the most common type of cancer among men, with approximately 1.4 million new cases diagnosed annually (1, 2). This number is projected to rise to 2.9 million by 2040 (3). While these figures are concerning, the prognosis for prostate cancer is generally favorable, with most patients expected to live for 15–20 years (4). According to 2022 statistics, there are approximately 8.3 million male cancer survivors in the United States, over 3.5 million of whom are prostate cancer survivors, accounting for 42% of all male cancer survivors (5). This population is expected to grow further in the future. While the primary goal of cancer treatment is often perceived as life prolongation, the emotional, physical, and financial burdens on patients are frequently overlooked. With the growing number of prostate cancer survivors, prioritizing their mental health becomes increasingly crucial.

It is noteworthy that prostate cancer patients have the highest risk of non-cancer related mortality compared to other cancer types (6). A study involving 1,643 men with localized prostate cancer showed that after a median follow-up of 15 years, only 2.7% of the patients died from prostate cancer, whereas a significant 19.1% died from other causes, including those who had never received treatment (4). Suicide is a significant cause of death among prostate cancer patients (7), potentially due to sexual dysfunction, gastrointestinal and urinary problems caused by the cancer and its treatment (8), as well as the severe psychological stress associated with a cancer diagnosis (9, 10). A Swedish study encompassing 180,189 prostate cancer patients found that the risk of severe depression and suicide in high-risk prostate cancer patients was significantly higher than that in the general population, with risk ratios of 1.82 and 2.43 respectively, persisting for over 10 years post-diagnosis (11). Moreover, a meta-analysis revealed that the crude suicide mortality rate among prostate cancer patients was 47.1 per 100,000 person-years, with nearly 10% of patients experiencing suicidal ideation (12). Another meta-analysis indicated that prostate cancer patients have a 1.46 times higher risk of suicide compared to the general population (13) The impact of suicide among patients is often more profound and unbearable for their families and healthcare providers than other causes of death. Although the absolute risk of suicide among prostate cancer survivors is lower compared to other causes of death, suicide deaths are preventable.

At present, there is a lack of models to guide clinical practitioners in predicting the suicide risk among individual prostate cancer survivors. Consequently, developing a model capable of effectively identifying high-risk groups for suicide among these survivors is crucial for prevention. Nomograms, which are visualization tools based on multivariate models, are commonly used to assist clinicians in evaluating different treatment options and determining disease prognosis (14, 15). This study utilizes the surveillance, epidemiology, and end results (SEER) database to construct a comprehensive nomogram designed to assess the individual suicide probabilities of prostate cancer patients. This nomogram assigns a corresponding score to each variable, and the X-tile software is then used to determine the optimal scoring cutoff values for distinguishing high-risk suicide groups. This tool will assist clinical practitioners in targeted preventive interventions.

## Materials and methods

### Patient selection and variables

The data utilized in this study were sourced from the SEER database (version 8.4.3), maintained by the National Cancer Institute (NCI). The SEER database covers approximately one-third of the U.S. population and provides detailed information on clinical pathology and survival outcomes. We extracted records of prostate cancer patients diagnosed between 2010 and 2017, collecting key clinical characteristics such as age, race, marital status, household income, histological type, pathological grade, PSA levels, Gleason score, TNM stage, as well as treatment modalities including surgical status, chemotherapy, and radiotherapy, along with survival outcomes. Marital status was categorized into married and unmarried, which includes single (never married), domestic partner, separated, widowed, and divorced. Households are categorized into two income groups: high-income households with an annual income of $65,000 or more, and low-income households with an annual income less than $65,000. The Gleason score was assessed from prostate biopsy samples. TNM staging was according to the 7th edition of the AJCC. The primary endpoint of interest is suicide, with survival time defined as the duration from the date of cancer diagnosis to the date of death by suicide. To ensure the accuracy and reliability of our study, we established strict inclusion and exclusion criteria. Inclusion criteria include: Patients diagnosed with prostate cancer as their only malignancy. Exclusion criteria include: Records containing any unknown variables in the data.

### Statistical analysis

First, we used X-tile software (version 3.6.1) to convert continuous variables into categorical variables, including age, household income, PSA levels, and Gleason Score. Subsequently, the entire cohort was randomly divided into a training cohort and a validation cohort at a ratio of 7:3. In the training cohort, preliminary variable selection was conducted using univariate Cox regression, best subset regression (BSR), and the least absolute shrinkage and selection operator (LASSO). In the univariate Cox model, factors were only considered for subsequent analysis if their *P*-values were less than 0.05. The BSR method evaluated all possible combinations of variables, selecting the final variables based on the maximization of the adjusted R ^2^-value. LASSO regression determined variable selection based on the lambda.1se criterion. The variables selected by these three methods were then included in a multivariate regression analysis using a stepwise backward regression approach, with the final selection of variables determined by the minimum Akaike Information Criterion (AIC), thus constructing the model. We compared the receiver operating characteristic (ROC) curves of the three models to identify the best model and used it to construct the nomogram. The discriminative ability of the nomogram was evaluated using the area under the ROC curve (AUC) and the Concordance Index (C-index). Additionally, calibration curves generated through bootstrap resampling (1,000 iterations) were used to assess the consistency between the nomogram’s predictions and the actual outcomes. The nomogram was used to calculate the total score for each patient, and the X-tile software was utilized to stratify risk, thus dividing patients into low- and high-risk groups. Subsequently, Kaplan-Meier survival curves were used to analyze the suicide risk between these two groups. The log-rank test was employed to statistically evaluate differences between the survival curves, verifying the significant disparity in suicide risk between the different risk groups. All statistical analyses were performed using R statistical software version 4.2.2.

## Results

### Baseline characteristics

**FIGURE 1:**
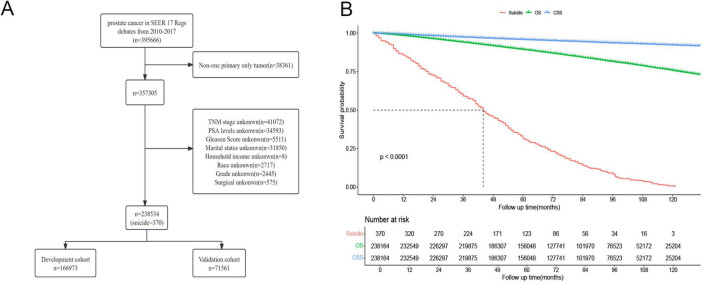
**(A)** Flow chart of prostate cancer inclusion and exclusion. **(B)** Survival analysis of prostate cancer patients who chose suicide versus those who did not.

This study encompassed data from 238,534 prostate cancer patients registered in the SEER database between 2010 and 2017, of whom 370 (approximately 0.16%) died by suicide (Figure 1A). Figure 1B illustrates that the 10-year Cancer-Specific Survival (CSS) rate and Overall Survival (OS) rate for prostate cancer patients were 92.3% (95% CI: 92.1–92.4%) and 75.7% (95% CI: 75.5–76.0%), respectively. The median overall survival time for prostate cancer survivors who chose suicide was 44 months (95% CI: 41–49 months). There were no significant differences between the training cohort (*n* = 166,973) and the validation cohort (*n* = 71,561) in terms of age, race, marital status, household income, histological type, pathological grade, PSA levels, Gleason score, TNM stage, surgical status, chemotherapy, radiotherapy, and suicide incidents (Table 1).

**TABLE 1:** Baseline clinical characteristics of prostate cancer patients.

Characteristics	Overall (%) *n* = 238,534	Training cohort (%) *n* = 166,973	Validation cohort (%) *n* = 71,561	*P*-value
Age				0.201
<65 years	110,781 (46.44)	77,745 (46.56)	33,036 (44.16)	
65–74 years	960,40 (40.26)	67,092 (40.18)	28,948 (40.45)	
≥75 years	31,713 (13.29)	22,136 (13.26)	9,577 (13.38)	
Race				0.899
White	186,577 (78.22)	130,591 (78.21)	55,986 (78.24)	
Black and other	51,957 (21.78)	36,382 (21.79)	15,575 (21.76)	
Grade				0.607
I	33,197 (13.92)	23,288 (13.95)	9,909 (13.85)	
II	101,473 (42.54)	70,927 (42.48)	30,546 (42.69)	
III	103,864 (43.54)	72,758 (43.57)	31,106 (43.47)	
Marital status				0.789
Married	178,453 (74.81)	124,890 (74.80)	53,563 (74.85)	
Unmarried	60,081 (25.19)	42,083 (25.20)	17,998 (25.15)	
Household income				0.100
Low	89,940 (37.71)	62,779 (37.60)	27,161 (37.96)	
High	148,594 (62.29)	104,194 (62.40)	44,400 (62.04)	
Histology				1.000
Adenocarcinoma	236,826 (99.28)	165,777 (99.28)	71,049 (99.28)	
Other	1,708 (0.72)	1,196 (0.72)	512 (0.72)	
PSA levels				0.682
<12 ng/mL	186,767 (78.30)	130,698 (78.27)	56,069 (78.35)	
≥12 ng/m*L*	51,767 (21.70)	36,275 (21.73)	15,492 (21.65)	
Gleason score				0.618
≤6	90,652 (38.00)	63,352 (37.94)	27,300 (38.15)	
7	97,777 (40.99)	68,530 (41.04)	29,247 (40.87)	
≥8	50,105 (21.01)	35,091 (21.02)	15,014 (20.98)	
T				0.107
1	101,236 (42.44)	70,770 (42.38)	30,466 (42.57)	
2	101,215 (42.43)	70,938 (42.48)	30,277 (42.31)	
3	33,675 (14.12)	23,629 (14.15)	10,046 (14.04)	
4	2,408 (1.01)	1,636 (0.98)	772 (1.08)	
N				0.671
0	228,773 (95.91)	160,121 (95.90)	68,652 (95.93)	
1	9,761 (4.09)	6,852 (4.10)	2,909 (4.07)	
M				0.840
0	229,002 (96.00)	160,310 (96.01)	68,692 (95.99)	
1	9,532 (4.00)	6,663 (3.99)	2,869 (4.01)	
Surgical status				0.514
No	134,102 (56.22)	93,798 (56.18)	40,304 (56.32)	
Yes	104,432 (43.78)	73,175 (43.82)	31,257 (43.68)	
Radiotherapy				0.627
No	152,716 (64.02)	106,848 (63.99)	45,868 (64.10)	
Yes	85,818 (35.98)	60,125 (36.01)	25,693 (35.90)	
Chemotherapy				0.075
No	236,148 (99.00)	165,343 (99.02)	70,805 (98.94)	
Yes	2,386 (1.00)	1,630 (0.98)	756 (1.06)	
Suicide				0.777
No	238,164 (99.84)	166,717 (99.85)	71,447 (99.84)	
Yes	370 (0.16)	256 (0.15)	114 (0.16)	

### Nomogram development and evaluation

**FIGURE 2:**
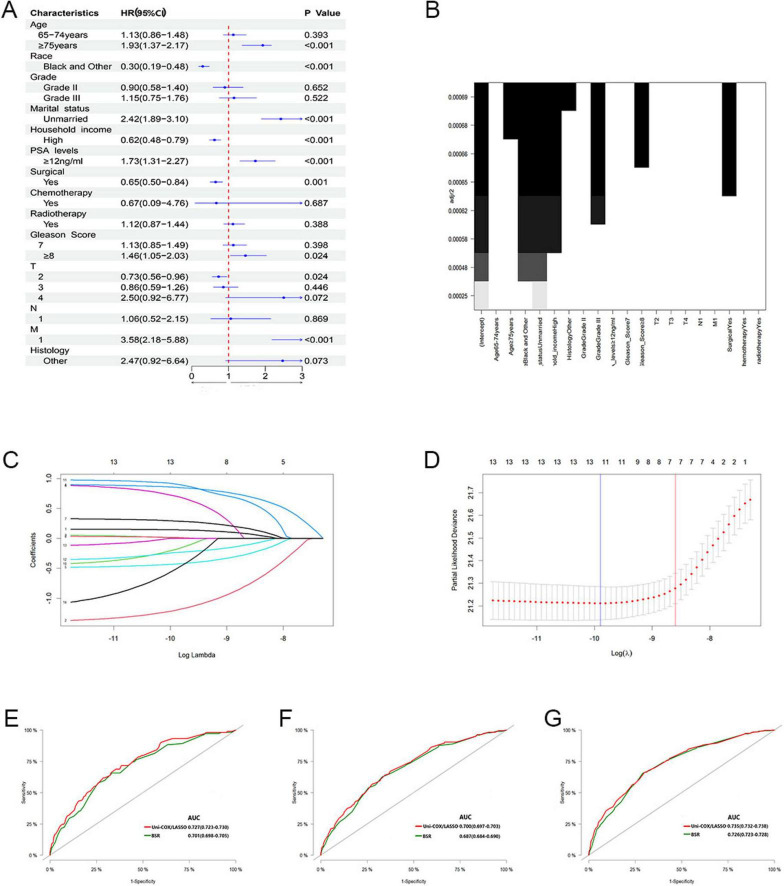
**(A)** Feature selection by univariate Cox regression. **(B)** Feature selection by BSR. **(C,D)** Feature selection by LASSO. **(E–G)** Comparison of 3, 5, and 10-year AUC values among three models.

**FIGURE 3:**
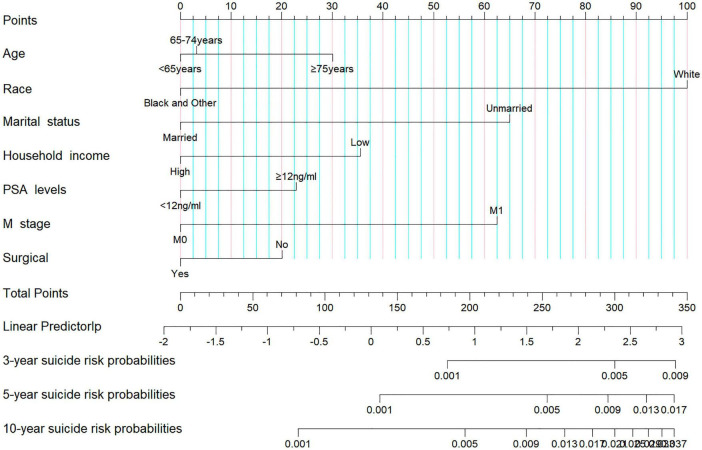
Nomogram for predicting the risk of suicide in prostate cancer survivors.

In our analysis, we began with 14 baseline variables: age, race, marital status, household income, histologic type, pathological grade, PSA levels, Gleason score, T stage, N stage, M stage, surgical status, chemotherapy, and radiotherapy. Through univariate Cox regression (*P* < 0.05) (Figure 2A), we identified nine significant variables: age, race, marital status, household income, PSA levels, Gleason score, T stage, M stage, and surgical status. Next, using BSR (Figure 2B), we selected eight variables based on the maximization of the adjusted R^2^ value: age, race, marital status, histologic type, pathological grade, household income, Gleason score, and surgical status. Through LASSO regression, determined by the lambda.1se value (Figures 2C,D), we identified seven variables: age, race, marital status, household income, PSA levels, M stage, and surgical status. The variables selected by each method were subsequently refined through backward stepwise regression analysis to determine the final variables for inclusion in the model (Table 2). Ultimately, univariate Cox regression and LASSO both selected the same seven variables (age, race, marital status, household income, PSA levels, M stage, and surgical status), with an AIC value of 5829.4. The BSR model finally selected six variables (age, race, marital status, household income, surgery, and histologic type), with an AIC value of 5841.9. Comparing the AUC values over 3 years (0.727 vs. 0.701), 5 years (0.700 vs. 0.687), and 10 years (0.735 vs. 0.726) based on these models (Figures 2E-G), the models constructed using univariate Cox and LASSO exhibited the lowest AIC and higher areas under the ROC curve. Therefore, we chose to construct the nomogram with variables of age, race, marital status, household income, PSA levels, M stage, and surgery (Figure 3). The model’s C-index of 0.702 indicates good discrimination ability.

**TABLE 2:** Final results of backward stepwise multivariate Cox analysis in three models.

Characteristics	Uni-Cox/LASSO		BSR	
	**HR (95%CI)**	***P*-value**	**HR (95%CI)**	***P*-value**
**Age**				
<65 years	Reference		Reference	
65–74 years	1.05 (0.79–1.37)	0.754	1.05 (0.79–1.38)	0.748
≥75 years	1.52 (1.06–2.18)	0.021	1.63 (1.15–2.33)	0.007
**Race**				
White	Reference		Reference	
Black and other	0.25 (0.15–0.40)	<0.001	0.25 (0.16–0.41)	<0.001
**Marital status**				
Married	Reference		Reference	
Unmarried	2.48 (1.93–3.19)	<0.001	2.56 (2.00–3.29)	<0.001
**Household income**				
Low	Reference		Reference	
High	0.61 (0.48–0.78)	<0.001	0.60 (0.47–0.77)	<0.001
**Histology**				
Adenocarcinoma			Reference	
Other			2.62 (0.97–7.04)	0.056
**PSA levels**				
<12 ng/mL	Reference			
≥12 ng/mL	1.38 (1.02–1.85)	0.036		
**M**				
0	Reference			
1	2.40 (1.41–4.08)	0.001		
**Surgical status**				
No	Reference		Reference	
Yes	0.76 (0.58–0.99)	0.041	0.71 (0.55–0.93)	0.013

### Nomogram validation

**FIGURE 4:**
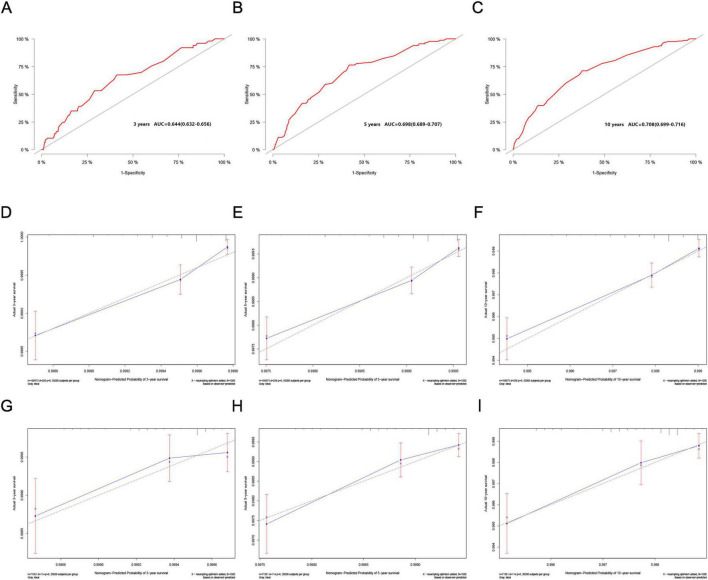
**(A–C)** ROC curves for the 3, 5, and 10-year nomograms on the validation cohort. **(D–F)** Training cohort and **(G–I)** validation cohort. Calibration curves for the 3, 5, and 10-year nomograms.

In the validation cohort, the nomogram demonstrated good discriminative ability. The C-index reached 0.688, with ROC curve values at 3, 5, and 10 years of 0.644 (95% CI: 0.632–0.656), 0.698 (95% CI: 0.689–0.707), and 0.708 (95% CI: 0.699–0.716) respectively (Figures 4A-C). As illustrated, calibration curves from both the training cohort (Figures 4D-F) and the validation cohort (Figures 4G–I) exhibited good calibration, indicating that the predicted probabilities of the nomogram closely align with the actual occurrence probabilities.

### Risk stratification analysis based on the nomogram

Utilizing the total scores calculated from the nomogram (Supplementary Table 1), we stratified patients into low- and high-risk groups using X-tile software. Patients in the low-risk group had total scores below 188, while those in the high-risk group had scores of 188 or higher. Cumulative probability curves for suicide occurrence among prostate cancer survivors indicated that the cumulative incidence of suicide was significantly higher in the high-risk group compared to the low-risk group in the training cohort (0.007 vs. 0.002, *P* < 0.001). This finding was also validated in the validation cohort (0.008 vs. 0.002, *P* < 0.001) and in the entire cohort (0.007 vs. 0.002, *P* < 0.001) (Supplementary Figure 1), confirming significant differences in cumulative suicide incidence between the two stratified risk groups.

## Discussion

Our study included 238,534 male prostate cancer patients, with a crude suicide mortality rate of 160 per 100,000 individuals. The nomogram effectively differentiates suicide risk among prostate cancer survivors, with the high-risk group having a 3.5 times higher risk than the low-risk group. Prostate cancer has traditionally been viewed as a “longevity” cancer, but suicide significantly reduces the life expectancy of these patients. Moreover, the act of suicide among prostate cancer survivors not only affects the individuals themselves but can also have a profound impact on their family members (such as spouses, parents, children), and may even prompt suicidal behavior among these close family members (16). Given these considerations, developing a model to predict the risk of suicide among prostate cancer survivors becomes particularly urgent.

Suicide risk among prostate cancer survivors is usually the result of the accumulation of multiple risk factors, rather than being reliably predicted by a single factor. Thus, identifying additional suicide risk factors and using multivariate models for risk assessment are considered more effective methods (17). In this study, variable selection was conducted using univariate Cox regression, BSR, LASSO regression, and backward stepwise multivariate Cox regression to reduce the risks of underfitting and overfitting. Ultimately, seven variables including age, race, marital status, household income, PSA levels, M stage, and surgical status were incorporated into the nomogram. Race, marital status, and M stage are the three most significant factors influencing suicide risk. A study involving 4.7 million cancer patients in the UK found that White cancer survivors had a significantly increased risk of suicide, a trend not observed in other racial groups (18). This racial difference may be associated with easier access to means of suicide, such as firearms among Whites, as one study analyzed 1,743 individuals who disclosed suicidal intentions, with 75% choosing firearms, of whom 96% were White (19). Additionally, research indicates that stronger religious beliefs, solid familial relationships, and greater psychological resilience may enhance individual resilience against suicidal pressures. These factors may be more prevalent in some Black communities (20, 21). Misclassification of suicide behavior among Black people could also be a factor (22). Marital relationships typically provide social support, emotional stability, and a sense of economic security, all of which help alleviate psychological burdens and thus reduce the risk of suicide (23). Research shows that individuals prone to disclose suicidal intentions are more likely to reveal these to their partner or close family members (19). Social isolation and loneliness, common among unmarried elderly living alone, are significant risk factors for suicide (24). Advanced tumor stages, such as distant metastasis and high PSA levels, have been proven to be associated with a higher risk of suicide (25–28).

To reduce the suicide risk among prostate cancer survivors, it is crucial to avoid overdiagnosis and overtreatment of prostate cancer, which can reduce the psychological stress and risk of treatment-related complications caused by cancer diagnosis and treatment. PSA screening is the most common method for detecting prostate cancer, but it has been controversial mainly because it often detects well-differentiated cancers that may not present clinical symptoms. Autopsy studies have shown that one-quarter of men aged 60–69 who died of other causes were found to have prostate cancer, and this proportion rises to one-third in men aged 70–79 (29). Although PSA screening has reduced the cancer-specific mortality risk of prostate cancer, its impact on overall mortality is not significant (30, 31). Over 60% of prostate cancer diagnoses occur in men over the age of 65, where the benefits of screening in reducing all-cause mortality are limited due to high competing risks of death and treatment-related complications in older men (32, 33). A study in Sweden found that low-risk prostate cancer patients identified through PSA screening had a significantly increased risk of suicide, adding to the all-cause mortality among prostate cancer patients (26). The US Preventive Services Task Force (USPSTF) recommends against routine PSA screening, although this may to some extent lead to an increased incidence of metastatic prostate cancer (34). This issue can be addressed by raising the PSA biopsy threshold, thereby reducing the diagnosis and overtreatment of clinically insignificant indolent prostate cancer (35). Studies have shown that for localized prostate cancer, active surveillance compared to active treatment shows no statistical difference in CSS and OS (4). However, patients in the active surveillance group experience fewer complications related to sexual function, intestines, and bladder (8), and also a lower risk of suicide (35). Therefore, future strategies should focus on identifying patients with potentially fatal prostate cancers to tailor treatments precisely, thus avoiding unnecessary psychological and physical burdens.

In addition to optimizing treatment strategies, addressing mental health is also crucial in reducing suicide risk. Suicide is often viewed as a stigma, and individuals at risk of suicide rarely seek help on their own. For prostate cancer survivors who are at high risk of suicide, particularly those with a history of suicide attempts, regular mental health screenings and interventions should be implemented, including psychotherapy and pharmacotherapy. Depression and anxiety are the most common psychological states in cancer patients, which are closely associated with suicidal behaviors (36, 37). Studies have shown that 70% of prostate cancer survivors who die by suicide suffer from depression (38). During the entire treatment process, the incidence of depression and anxiety in prostate cancer patients can be as high as 17.27 and 27.4%, respectively (9). The American Cancer Society recommends that prostate cancer survivors undergo a psychological health screening at least once a year (39). Various scales can help identify patients with anxiety, depression, and suicidal intentions. The Dana-Farber Cancer Institute assesses the psychological status of prostate cancer survivors through mail follow-up using a brief eight-item scale to evaluate recent suicidal thoughts, intentions, and behaviors, which is more acceptable to patients compared to face-to-face surveys (40). The Expanded Prostate Cancer Index Composite for Clinical Practice (EPIC-CP) is a scale tool that assesses urinary, bowel, sexual functions, and hormonal symptoms, efficiently and accurately evaluating the Health-Related Quality of Life (HRQoL) of prostate cancer patients. Nearly 80% of patients can complete this scale within 5 min, and nearly 90% of clinicians find it convenient to use (41). The 36-item Effects of Prostate Cancer upon Lifestyle Questionnaire (EPCLQ) assesses the impact of lifestyle changes after diagnosis and treatment of prostate cancer on anxiety and depression, where adverse emotions, social withdrawal, and plus loss of cognitive ability are key predictors (42). The PHQ-9 is a commonly used depression assessment scale (43), while the Hospital Anxiety and Depression Scale Depression subscale (HADS-D) and Self-rating Depression Scale (SDS) may underestimate the depression levels in prostate cancer patients (44).

Furthermore, limiting access to means of suicide plays a critical role in suicide prevention, especially in cases of impulsive suicide. Firearms, pesticides, and certain medications are the most common means of suicide (45). In different countries and regions, strategies for restricting access to means of suicide vary: in high-income countries, firearm suicide is more common (46, 47), while in middle- and low-income countries, pesticide suicide is more prevalent (48, 49). Among drug-induced suicides, benzodiazepines are the most common, followed by antipsychotics, highlighting the importance of strengthening the management of prescription medications (50).

This study has several limitations. First, the accuracy of death cause records may be compromised, particularly for suicide deaths, which can be confused with other accidental deaths. Second, the study did not include some important clinical features such as HRQoL scores and underlying conditions (including psychological disorders and other serious comorbidities), which are key factors influencing the risk of suicide in patients. Additionally, there is a lack of specific information about suicidal behaviors, such as records of suicidal intent and suicide attempts. Lastly, the predictive model was only internally validated, lacking external validation. Further independent cohort external validation would help ensure the robustness and generalizability of the model.

## Conclusion

Prostate cancer survivors constitute the largest group of cancer survivors globally, with suicide being one of the leading causes of death within this group. In response, we developed a simple and reliable predictive model based on easily obtainable clinical predictors. In response, we developed a simple and reliable predictive model based on easily accessible clinical factors, including age, race, marital status, household income, PSA levels, M stage, and surgical status. This model empowers medical professionals to quickly identify prostate cancer survivors who are at high risk of suicide and implement timely preventive measures. To further reduce the suicide risk among prostate cancer survivors, we propose three strategies: avoiding overdiagnosis and overtreatment, regularly conducting psychological health screenings and interventions for high-risk patients, and restricting access to common means of suicide.

## Data Availability

The original contributions presented in the study are included in the article/Supplementary material, further inquiries can be directed to the corresponding authors.

## Supplementary material

Supplementary Figure 1 Training cohort **(A)**, validation cohort **(B)**, and entire cohort **(C)**: suicide risk scenarios among prostate cancer survivors in low- and high-risk groups.
