# Supplementary material for: Developing a model for predicting suicide risk among prostate cancer survivors
Source: Front Med (Lausanne). 2025 Apr 10;12:1483266. doi: 10.3389/fmed.2025.1483266 (PMC12018404; doi:10.3389/fmed.2025.1483266)
Supplement: Supplementary file 1 [file Table_1.docx]

Table S1. Score of each variable in nomogram.

| characteristics | score |
| --- | --- |
| Age |  |
| <65years | 0 |
| 65-74years | 3 |
| ≥75years | 30 |
| Race |  |
| White | 100 |
| Black and Other | 0 |
| Marital status |  |
| Married | 0 |
| Unmarried | 65 |
| Household income |  |
| High | 0 |
| Low | 35 |
| PSA levels |  |
| <12ng/ml | 0 |
| ≥12ng/ml | 23 |
| M |  |
| 0 | 0 |
| 1 | 63 |
| Surgical status |  |
| No | 20 |
| Yes | 0 |
| Low-risk group | ≤186 |
| High-risk group | ≥188 |
