# Supplementary figures and images for: Developing a model for predicting suicide risk among prostate cancer survivors
Source: Front Med (Lausanne). 2025 Apr 10;12:1483266. doi: 10.3389/fmed.2025.1483266 (PMC12018404; doi:10.3389/fmed.2025.1483266)

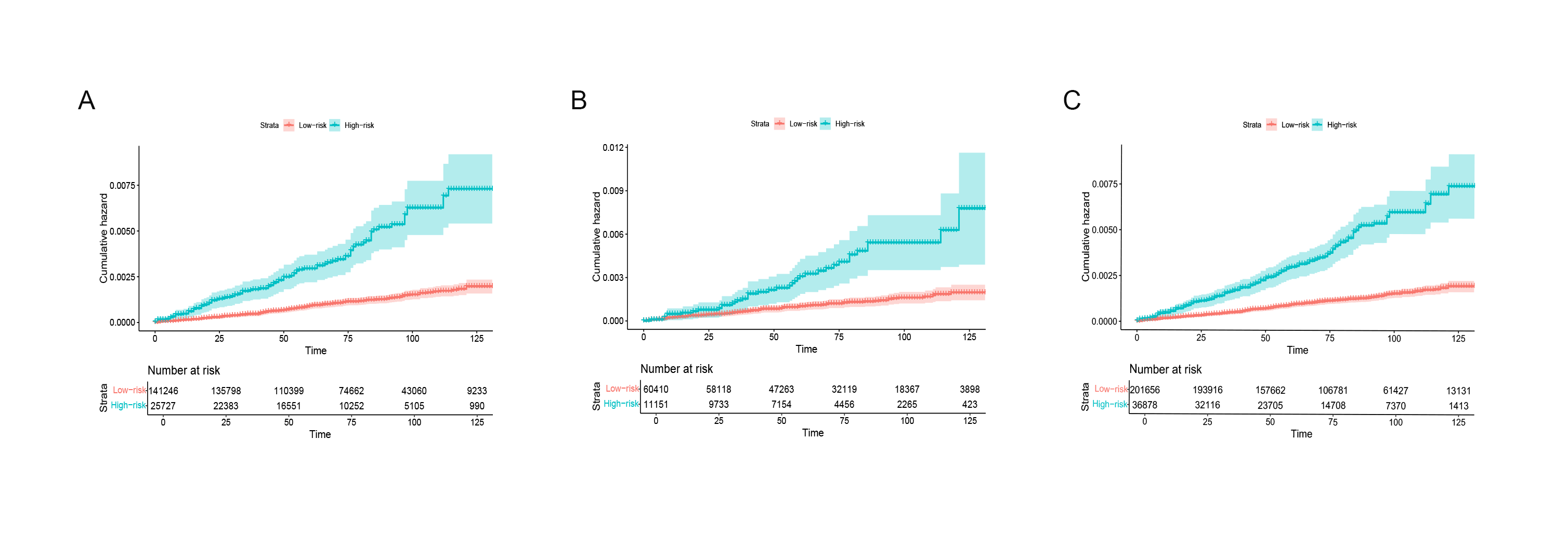

Supplement: Supplementary Figure 1 — Training cohort (A), validation cohort (B), and entire cohort (C): suicide risk scenarios among prostate cancer survivors in low- and high-risk groups. [file Image_1.tif]
